# Care workers, the unacknowledged persons in person-centred care: A secondary qualitative analysis of UK care home staff interviews

**DOI:** 10.1371/journal.pone.0200031

**Published:** 2018-07-09

**Authors:** Adam Kadri, Penny Rapaport, Gill Livingston, Claudia Cooper, Sarah Robertson, Paul Higgs

**Affiliations:** 1 UCL Department of Old Age Psychiatry, Division of Psychiatry, University College London, London, United Kingdom; 2 Camden and Islington NHS Foundation trust, London, United Kingdom; University of Antwerp, BELGIUM

## Abstract

Personhood discourses in dementia care have gained prominence and current care home standards mandate that care should be “person-centred”. However, it is unclear how the personhood of staff is construed within the care relationship. This paper aims to explore how the personhood of paid carers of people with dementia can be understood by focussing on the views and experiences of care home staff. We undertook a secondary qualitative analysis of interviews with 25 paid care staff in England, conducted as part of the MARQUE (Managing Agitation and Raising QUality of lifE) study. The authors inductively developed themes around the topic of personhood for staff, contrasting management and care staff perspectives. We found that many care staff are not identified as persons in their own right by their employing institutions, and that there is a general lack of acknowledgment of the moral work of caring that occurs within formal care work. This oversight can reduce the complex relationships of care work to a series of care tasks, challenges care workers’ self-worth and self-efficacy, and impede their efforts to deliver person-centred care. We conclude that care staff status as persons in their own right should be explicitly considered in quality standards and supported by employers’ policies and practices, not simply for their role in preserving the personhood of people with dementia but for their own sense of valued personhood. Enhancing staff personhood may also result in improved care.

## Introduction

### Personhood in dementia and person-centred care

Personhood in dementia care is increasingly important within the academic and professional care literature, including healthcare policy and practice [1, 2]. Much of the current understanding of personhood in dementia originated from Kitwood’s work. He defined personhood as, “a standing or a status that is bestowed on one human being, by another in the context of relationship and social being” [3]. This marked an important shift in dementia discourse; away from a purely disease model, to one incorporating the importance of psychosocial elements: a person’s history, and their social interactions and perceptions [1]. United Kingdom care quality standards moved to mandating that those receiving care should be treated in a person-centred and dignified manner, as a person rather than an illness [4]. While the term ‘personhood’ can be seen as problematic [5], it can be argued that using it to reframe discussions about dementia has helped to reduce stigma, improve care and promote the citizenship rights of those living with dementia [6].

Kitwood’s model highlighted the relational components of personhood, engaging both ‘cared for’ and ‘carer’ in its construction and maintenance. He suggested that good care practices treat and respect people with dementia as deserving of appropriate social interaction. Bad care practices create a ‘malign social psychology’ and seek to undermine their personhood by denying them social involvement [3]. Despite the irreversible neuropathological changes in dementia, person-centred care and psychosocial interventions that develop and maintain social relationships can help mitigate disability. This is achieved by reducing agitation and other neuropsychiatric symptoms, and improving quality of life [7, 8]. Close family bonds and professional care relationships are important in sustaining the personhood of people with dementia and enhancing care quality [9, 10], so a sociologically informed perspective on personhood in dementia care is important [11]. Previous authors have called for more relationship-focused care, focussed on maintaining the wellbeing of family carers as well as people with dementia [12, 13].

### The importance of paid care staff

Paid care staff are critical to delivering programmes of person-centred care to many people with dementia, especially those who live in care homes. A recent review of the effectiveness of institutional, person-centred care strategies found that investing in the learning and skill development of care staff was crucial to their successful implementation, and the sustainability of the effects on behavioural problems [7]. Other studies have shown that relationships within care homes are key determinants of the effectiveness of interventions, which are less likely to be implemented where staff experience low morale and feel undervalued, and homes are understaffed [14–16].

Kitwood acknowledged the importance of maintaining staff wellbeing [3] for the effective care of residents. Some reviews have considered the effects of person-centred care interventions on dementia care workers. The variability of studies makes it difficult to draw conclusions [17]; though one review described positive impacts on staff job satisfaction and capacity to meet client’s needs in a constructive way [18]. The culture change movement in the USA [19], aimed at enhancing person-centred care across the care sector in a holistic manner, has seen increased emphasis placed on developing staff well-being, communication and interactions between staff and residents [20, 21]. Higher job satisfaction has been associated with lower burnout, higher global empowerment, higher organisational support, higher psychological empowerment, stronger work group cohesion and higher personal accomplishment [22].

### The absent issue of the personhood of care staff

The research and debate on the subject of paid carers has primarily focussed on improving quality of care, costs and turnover [23, 24]. In the UK, and other European countries, staff who work in care homes are generally low paid, poorly trained, and work in conditions of high pressure with staff shortages and high staff turnover [25, 26]. While some care staff, for example nurses, hold professional qualifications, many do not. Research about family carers of older people has often centred on burden and the stress of caring [27, 28] and whilst these have also been studied for paid carers, evidence in this area has typically been sparse and often of low quality [29, 30]. Higher staff burnout has been associated with less support and higher job demands [31].

Although person-centred cultures have led to increased interest in staff skills, relationships and conditions, including a concern with issues of stress and burden, it seems that the motivation is often to improve productivity and retention. We suggest it is a moot point that these studies generally consider the personhood of staff mainly as facilitators (or instruments of production) of enhanced resident personhood, rather than being of intrinsic value in itself. This point has been made by Higgs and Gilleard in a book 'Personhood, identity and care in advanced old age' where they point to the fact that the 'unskilled' status of much care work means that many workers are both undervalued and expected to carry out the 'moral work' associated with the imperative to care [32]. Discourses around personhood typically focus on that of the person with dementia, with the issue of the personhood of their paid carers being largely absent. Given that personhood is a status attributable to all individuals, it should be asked, where is the personhood of staff? We argue that to be consistent with a philosophy of person-centred care in dementia, this intrinsic aspect of care staff needs to be recognised.

## Materials and methods

### Ethics

National Research Ethics Service (NRES) committee London—Queen’s Square gave ethical approval for the study; reference number 14/ L0/ 0697.

### Study background and aims

As part of the MARQUE study: Managing Agitation and Raising QUality of lifE [33], we conducted interviews to investigate how care home staff understand and manage agitation in people with dementia as part of person-centred care [34]. During the analysis, we found a secondary theme relating to an unacknowledged personhood of staff. This theme was not the study’s original aim. These secondary findings pointed to care staff feeling unacknowledged as part of the implementation of person-centred care. This seemed to go beyond poor staff relations and work conditions, as staff respondents reflected upon dealing with complex issues of diagnosis and person-centeredness.

Our aim is to develop and present these findings. By considering rich personal experiences of staff working in dementia care, we aim to understand whether the personhood of staff within person-centred care is also being acknowledged.

### Design and sample

This is a secondary analysis of interviews of UK care home staff, conducted in 2014 and 2015. The primary, inductive thematic analysis investigated how care home staff understand and manage agitation in people with dementia [34].

Care homes and staff were purposively sampled for maximum variation to include a mixture of care home sizes, types (residential or nursing) and locations (urban and rural areas); and individual staff demographics (sex, age, ethnicity, nationality), roles and levels of experience. Staff characteristics are described in Table 1.

**Table 1:** Care staff socio-demographic and employment characteristics.

Socio-demographic	Category	Care staff n (%)
Sex	Female	17 (68)
	Male	8 (32)
Ethnicity	Asian British	1 (4)
	Asian or Asian British: Bangladeshi	1 (4)
	Asian or Asian British Indian	4 (16
	Black or Black British African	3 (12)
	Black or Black British Caribbean	3 (12)
	White British	6 (24
	White other	5 (20)
	Mixed white and Asian	1 (4)
	Other—Mixed Caribbean	1 (4)
English as first language	No	13 (52)
	Yes	11 (44)
	Not known	1 (4)
Staff role	Care assistant (care staff)	9 (36)
	Manager / deputy manager (management)	5 (20)
	Team leader (care staff)	7 (28)
	Activities coordinator (care staff)	2 (8)
	Nurse (care staff)	2 (8)
Shift pattern	Days	18 (72)
	Days and nights	7 (28)
Length of service	Less than 1 year	4 (16)
	1 to 5 years	13 (52)
	6 to 10 years	8 (32)
Nursing qualification	Yes	10 (40)
	No	15 (60)

Staff were interviewed from 6 homes (3 residential, 3 nursing). Nursing home definitions vary by country. Referencing international definitions, the homes sampled from were all, “(a) facility that provides room and board and … assistance with activities of daily living in patients who are physically and/or cognitively impaired. Typically, …. medical professionals are not available on site.” [35] The nursing homes had nurses on site. According to the Care Quality Commission (CQC; the independent regulator of all health and social care services in England), all the homes offered specialist support to people with dementia. At their most recent routine CQC inspection prior to participating in the study, four of the six homes met all assessed quality standards, one required action on three out of five quality standards and one required action on one of seven quality standards. All homes subscribed to person-centred care approaches.

Staff gave written informed consent to be interviewed. PR (clinical psychologist) individually interviewed 25 care home staff working in the six care homes in and around London, UK. Interviews took place in private rooms within the care homes. PR used a semi-structured interview schedule, based on the research literature, consultation with family carers of people with dementia and research team expert opinion. The questions focused on understanding how staff conceptualise and cope with agitated behaviours of residents within the context of their work environment. Open-ended questions were revised iteratively to explore emerging issues.

Interviews were audio-recorded and transcribed verbatim. Interviews were coded using NVivo software. The primary thematic analysis was conducted by PR and independent raters.

### Analysis

#### Secondary data analysis

The secondary use of data and data sharing more generally can be of great value to scientific understanding and progress [36] and this approach has seen a rapid growth [37]. This study adopts the approach of reanalysing a data set by focusing on a concept that seemed to be present but was not specifically addressed in the primary analysis, leading to the generation of new knowledge and greater utility of the original data set [38]. Such approaches can have particular utility in addressing a sensitive area of research, which may otherwise have no have been feasible [39].

It is recommended that when doing a secondary qualitative analyses, the research questions for the secondary analysis be sufficiently close to those of the primary research and that the data collection and analytic techniques in the primary dataset are similar to those that will be applied in the secondary analysis [39].

For the current study, we re-analysed all interviews. The first author (AK) immersed himself in the original recordings and transcripts to obtain a rich understanding of the content. We inductively developed themes around the topic of staff personhood, according to the literature on personhood and care practices, as well as discussions with co-authors.

The term ‘personhood’ has been the subject of much discussion. However, most agree with Kitwood's emphasis on maintaining the personhood of care home residents with dementia by showing attention and respect [40]. For our analysis, we were interested in investigating whether this also applied to the care home staff who provide this care.

We considered and contrasted both management and care staff views. For this purpose, management views comprised of those by care home managers or deputy managers. All other staff were grouped as care staff (see Table 1).

Whilst those with nursing qualifications may have different levels of training, experience and knowledge that sets them apart from other paid care staff, this is often a complex picture. A previous study found that those employed as nurses often have all-embracing roles within the home, and thus have difficulties defining and limiting their roles. It is suggested that this ambiguity can lead to an increased sense of responsibility and difficulties in task designation [41] and many job descriptions may not clearly define the roles and responsibilities of nurses and care assistants respectively.

In addition, many of those staff who have nursing qualifications from their home countries are unable to practice with them in the UK. Whilst 10 staff interviewed reported as having nursing qualifications, only two staff members were employed as nurses. In contrast, four of the five management staff had nursing qualifications. Four care assistants/senior carers who reported having a nursing qualification did not state if they were currently registered in the UK or what type of nursing qualification they had. However, they were not employed as nurses in their respective institutions. The two individuals employed as nurses worked within nursing homes but were not acting as managers or were working across the home. For these reasons, we allocated them to the care staff category during our analysis. However, it was important to consider if their views differed to those presented within the care staff data. Any notable differences were considered and drawn out separately. Those viewpoints which explicitly addressed the difference between nurse and other care staff views were also considered.

We developed themes relating to the personhood of staff using a process of adaptive theory [42]. As themes were drawn out and developed, we adapted our theory accordingly. All processes were discussed by the authors in regular meetings to ensure rigor.

#### Analysis step 1

Drawing on the theoretical context, we inductively developed themes around staff personhood by initially considering:

1. How staff and management describe the challenges of providing person-centred care for people with dementia.
- These were subsequently grouped into categories of issues relating to dementia care, and those relating to care organisation.
2. The ways in which care staff are viewed and acknowledged (by themselves and others), particularly as being persons in their own right, deserving of dignity and respect.
- These were subsequently grouped into categories relating to the identity of care staff, and views of the care role.

The resulting separated analyses of interviews from management and care staff produced a number of inductively developed primary themes relating to these initial foci. As primary themes were drawn out, they were refined and sub-themes identified, through a process of constant comparison. These were grouped within comparable categories: see Tables 2 and 3.

**Table 2:** Management primary themes and categories.

Management primary themes and categories				
*Categories*	Care staff delivering PCC	Organisational	Identity of care staff	Views of care role
*Themes*	Staff viewed as incompetent in their work	Manager’s levels of support for staff	Backgrounds of care staff	Role differences between management and care staff
	Staff abilities to handle agitation	Staff training issues	Personalities of staff	Staff level of investment in the job: idea of ‘vocation’
	Integrating task vs person-oriented care	Team problems and cultures of staff	Positive and negative views of staff	
	Appreciations of the difficulties for staff	Care home organisational and management restrictions	Views of the value of Staff	

**Table 3:** Care staff primary themes and categories.

Care staff primary themes and categories				
*Categories*	Delivering PCC: issues related to dementia	Delivering PCC: issues related to organisation	Identity of care staff	Views of care role
*Themes*	Staff dealing with people with dementia’s agency, identity, rationality	Levels of support and understanding from management	Staff attitudes towards caring	Feeling disillusioned
	The co-construction of care	Responsibilities and unrealistic demands	Personalities and cultural differences of staff	Staff views of their value
	Dirty work	Effects of organisational pressures on PCC		Levels of investment in role
	Impact of agitation on staff, including ‘abuse’	Staff views on organisation		Issues related to role differences
	Working with resident’s relatives	Issues within the team		
	Emotional burden of caring	Integrating task vs person-oriented care		

#### Analysis step 2

We contrasted management and care staff primary themes and content to produce 8 main themes, considering the overall implications of contrasted viewpoints. This was done through a process of constant comparison and discussion between authors. These are described in Table 4. An additional focus that emerged in this stage concerned whether staff are perceived solely in an instrumental fashion to enhance resident personhood, or whether they can too be acknowledged for their intrinsic role as fellow 'persons'?

**Table 4:** Contrasted main themes and categories.

Contrasted main themes and categories				
*Grouped categories*	Delivering PCC	Organisational issues	Identity of care staff	Views of care role
*Main themes*	Difficulties of caring for people with dementia unacknowledged e.g. agitation/’abuse’	Support and understanding often inadequate	Personal identity and experiences not respected	Staff seen as instruments of care
	Difficulties of person-centred vs task oriented care unacknowledged	Organisational pressures make caring difficult, out of control for carers		Feeling out of control and unimportant
	Burden of care work unacknowledged			

#### Analysis step 3

We considered further the contrasted main themes, adapting into theory and then consolidating these into overarching themes. Overall we developed two overarching themes relating to staff personhood. These are the themes that summarise the overall implications of the content.

For example, main themes relating to the delivering of person-centred care suggested an overall lack of acknowledgment of the difficulties care staff face; and, where these difficulties undermined the personhood of staff, such as in agitation/’abuse’ and lack of support, these were grouped into the overarching theme: ‘Care staff not accepted as dignified persons in their own right’. Main themes, ‘Personal identity and experiences not respected’ and ‘Staff seen as instruments of care’ were accordingly also part of this theme.

Those main themes and content that related to an instrumental approach to delivering care, such as task-oriented versus person-centred practices and organisational pressures and their effects on care staff, including care staff and management responses were grouped into the theme, ‘Care staff’ valuation of themselves as well as by others alongside subsequent job satisfaction were diminished through conflicting demands, organisational pressures and a lack of control.’ The content of these overarching themes are described below.

## Results

Overall we developed two overarching themes relating to the personhood of staff.

### Care staff were not accepted as dignified persons in their own right

- Staff were not thought of as being subjected to abuse
- Needs for respite from emotional burden and support unmet
- Care staff seen as the instruments of care—lack of respect for personal identity and experiences

### Care staff valuation of themselves as well as by others alongside subsequent job satisfaction were diminished through conflicting demands, organisational pressures and a lack of control

- Staff caught in conflicts between person-centred work and a task-oriented system
- Feeling out of control and unimportant
- Management and care staff’ response to conflicting demands and pressures

### Care staff were not accepted as dignified persons in their own right

Whilst care staff reported practising principles of person-centred care, they often spoke of how they themselves were not treated in a similarly dignified manner. Findings revealed situations where the complexities of implementing person-centred care were underappreciated, and staff vulnerabilities and needs were left unacknowledged. There was a sense that in order to maintain the personhood of people with dementia in these conditions, the personhood of the staff was diminished: that staff were expected to subjugate their own personhood in order to maintain the personhood of residents:

“*Everybody is human and you can only take so much of someone shouting the same thing at you over and over again.” (Care assistant 1)*

### Staff were not thought of as being subjected to abuse

Some staff expressed frustration that verbal and physical aggression they experienced when a resident was agitated was neither acknowledged nor led to action. Some staff considered this a form of abuse. However, one repeated theme was that such behaviour was an unavoidable and necessary part of the job and had to be accepted:

“*Practically, we receive all the abuse, you know, and we only have to look after one… the thing. That’s only from our perspective of course. I mean, considering then they are vulnerable, it’s fine. I can take the abuse. I don’t mind. I don’t care. In the end I have to do it, my dear, because otherwise I can’t work tomorrow.” (Care assistant 2)*

Another carer spoke of the complexities of handling such situations and how they could not show their fear or anger as this might escalate things:

“*[…]* *a big part of it is the staff need to be very relaxed*. *They can’t let it get to them*. *As soon as*, *you know*, *carers are lashed out to*, *they have their hair pulled*, *or are scratched*, *things like that*, *they need to keep their cool*, *because that reaction to it can have the biggest impact*.*” (Care Assistant 3)*

This care assistant also described how little sympathy would be shown for staff being ‘abused’, from within the home and by the outside world:

“*Sometimes it would be so lovely to hear a nice story about dementia, and staff, and what people do, and also it feels like, you know, you don’t hear things about residents lashing out at carers.[…] My friend works in a higher, severe unit, you know, and her colleague got smashed with a rock on the back on the head. You know, that wouldn’t come out, because residents wouldn’t do that to the staff, would they?” (Care assistant 3)*

Some care home managers were explicit that care staff could not be seen as victims of abuse from those with dementia:

“*When, even… I was called all the time, that who hit who, or who scratched, you know. Instead of managing the situation, they were reporting and they expected someone else to come and resolve the problem.” (Home manager 1)*

### Needs for respite from emotional burden and support unmet

Many staff spoke of how the emotional burden of caring for people with dementia affected them both at work and at home and they needed time for rest and reflection:

“*When you walk out them doors you need to shut it off […] You know*, *if you’ve got a really verbally abusive resident […] sometimes carers feel it can affect them outside*, *if they can’t switch off*. *I know one carer who left because she used to have dreams about residents*, *and I think that coping*, *turning that switch off*, *as well*, *is really important*.*” (Care assistant 3)*

However, management rarely acknowledged this need in staff. One deputy manager spoke of their frustration about carers taking a break away from residents:

“*[…] here what I see is that sometimes they, kind of, separate… you know […] Now I’m on break, I’m going to go as far away as… from here as I possibly can, which, yes, it’s not ideal. I can understand it. From a selfish point of view, I can understand it. If you work for 12 hours you want to have ten minutes to yourself, so in a way I can understand it, but it’s… yes, it would be better the other way.” (Deputy manager 1)*

Many staff were dissatisfied with support from their managers and felt that they were not looked after, but that their only support was from each other. These issues were felt by those throughout the seniority scale:

“*Just the lack of support, because there’s nobody coming up to you and saying, how was it this morning, or how was your day? And I think if you go through the whole building, talking to the nurses, you’ll find, you’ll hear the same story. They don’t seem to think that, you know, just to show themselves and to come and just say, are you managing? And when you’re short of staff it’s just, get on with it, you know, and especially the times when you’re really stretched and you’ve got lots going on, and then, that’s it, nobody even looks to say you’ve coped, you know.” (Nurse unit manager 1)*

“*Yes, I think if we're suffering from stress we're meant to ring a number for example, but I'm not going to go down that route. It's just helping your colleague really, if your colleague's going to get behind you just step in and help them really.” (Care assistant 4)*

However, this was not universal. A simple acknowledgment of their role was valued:

“*Yeah you do get times when you feel like that but as I say over the last three or four months where things have settled we’ve got a new suite manager and we’ve got our sort of team now the suite manager will say at the end of the evening thank you very much or will even sort of like say my name or whatever as she’s going through and it does make a difference, you feel like you’ve done a good job.” (Care assistant 1)*

### Care staff seen as the instruments of care—lack of respect for personal identity and experiences

A respect for personal identity is considered integral to person-centred care, and many carers spoke of valuing caring and how this originated from their own backgrounds:

“*I’m doing this job for fifteen years, and I enjoy being a carer, because my grandma was, she had dementia as well and I know it’s difficult, to have, for the people to have, this kind of, we call it illness. Before I came here, my grandmother had this illness… Back home, we don’t have these kinds of old people’s homes, so it’s just the family who looks after them, so it’s very difficult.” (Care assistant 5)*

However, it seemed that these values and expertise were often not appreciated or fully understood. Managers spoke of staff backgrounds as a problem rather than an asset and of difficulties with foreign and more experienced staff adapting to preferred attitudes or skills. It seemed that their personal caring-related values were ignored, and instead they were seen more as the instruments for operationalising care routines. This manager described their experience of finding carers “hard to change”:

“*We’ve got people who used to be nurses and things like that from other countries, and it’s very hard to instruct them on what to do. Even if they clearly understand, it’s very hard to instruct them or change their ideas on what is the right way to be and, I mean, that’s not only an immigrant problem. You find that with the more experienced staff that it’s hard to change, so there is a culture of change that’s really, really hard to break down.” (Deputy manager 2)*

It seemed relatives could also see staff as proxies to operationalise what they felt was good care or right for the resident, without consideration of a carer’s relationship, experience and knowledge of the residents, and ultimately their own personhood:

“*It’s just sometimes they will just tell you*, *oh*, *do this*, *do that*, *you know*, *or my mum doesn’t want to do this or my dad doesn’t want to do this*. *But we just deal with it*.*” (Care assistant 6)*

“*It can make you feel you’re not doing your job but then also it can annoy […] I’m here for that resident, that person who is here and not for you. So don’t make his or her life uncomfortable by insisting that they should sit in a chair all day in the TV lounge or something.” (Care assistant 1)*

This carer also spoke of how the negative reports in the media could lead to further objectification of a carer’s role in the eyes of relatives:

“*I find that quite distressing because I think, you know, just because people have done stuff like that it’s like you’re being tarred with the same brush, that it’s happening to their loved ones and I do find that quite upsetting.” (Care assistant 1)*

### Care staff valuation of themselves as well as by others alongside subsequent job satisfaction were diminished through conflicting demands, organisational pressures and a lack of control

Many carers spoke passionately about delivering the ideals of person-centred care, often citing their own personal desires and duties to look after residents who they saw as deserving of care. They strove to help maintain the personhood of residents, including their social interactions and abilities to participate in care. When the task system and institutional constraints made this work difficult or impossible, they were often forced into an instrumental form of care and there was a real sense that the carer’s personhood, their identity as a good carer, was challenged.

### Staff caught in conflicts between person-centred work and a task-oriented system

This care assistant spoke of the importance of compassion and their perceived duty to care:

“*Compassion, for me that is very important; you have to understand that this certain person is having a problem, is having a health problem, and if he's agitated he's agitated for a reason, you have to understand that he can't help it and you have to deal as nicely as possible and you have to have compassion for him, you have to help him as much as possible.” (Care assistant 7)*

Care staff often felt that their desire to care for people with dementia was threatened by care delivery processes. They felt conflicted between a desire for what they understood as person-centred care and a task-oriented system, and often between the imperative to keep someone safe and to allow them to refuse care. This carer’s comments reflect the difficulties of practising person-centred care with these constraints:

“*I’ve said, you know, don’t feel the pressures of care, because there’s so many pressures with care, like getting things done by a certain time, especially with personal care. Especially if that resident is in a wet bed, they’ve taken their pad off, or anything like that, you know, you feel the pressure of people above you saying, do the personal care because if the family come in and see them like that they’re going to think it’s neglect. But, actually, it’s more neglect doing it without the person’s consent.” (Care assistant 3)*

#### Feeling out of control and unimportant

Staff discussed the demoralising effects of practising person-centred care within organisational structures over which they had no control. There were many perceived pressures including: long shifts, poor work and employment conditions, low respect, unsuitable environments, bureaucracy, low staffing levels, profit driven organisations, poor regulation of the profession, and the effects of the media. At worst these pressures resulted in ethical dilemmas for staff, forcing them to make difficult decisions, the outcome of which they felt held accountable for.

This nurse spoke of the frustration of such constraints and how it affected their ability to care effectively:

“*When we talk about staffing, we’re just told, no, you can’t have any more staff […] in the evening, we can’t do our jobs because the carers are going around together doing personal care, they’re behind closed doors. We’ve got three, four residents in the corridor, they could be hitting, they could be pushing, there could be people falling over. So, we have to leave our jobs and we go into the corridor to make sure people are safe or in the lounge, but nobody listens to that. They just think, oh, you know, you can’t have any more staff and that’s it. So, it is very stressful.” (Nurse unit manager 1)*

Some carers were explicit about the contradiction between a care company’s person-centred care rhetoric and a reality where business dictates decisions:

“*Well they set their own particular standards and everything and an organisation either looks after you or it doesn't really. There are a lot of words and intentions and propaganda but when you start stripping, going beneath the veneer on that propaganda it's a bit disappointing to me really. I think it's all about profit, that sort of thing.” (Care assistant 4)*

#### Management and care staff response to conflicting demands and pressures

Whilst management promoted person-centred care and had concerns that staff were often too task oriented, the overall expectation seemed to be that staff should be able to balance understanding and caring for people within a procedural system without either care component suffering. More emotionally demanding skills such as managing agitation and maintaining close relationships with residents were seen as part of the same task system of care, and their inherent demands were not necessarily appreciated. There was an assumption that if staff remained compassionate, patient and flexible, they could resolve difficult situations. Responsibility was left to individual staff members, rather than considered at an organisational or team level.

This manager described how such difficult work should be manageable:

“*I think there's a bit of a confrontation and some people understand it and do it. So you know you've got to get things done, making the beds, keeping the place clean, tidying the wardrobes and all that sort of stuff and other people… but still see the client, still looking out for the client, still caring but they're getting other things done at the same time.” (Home manager 2)*

Many care staff and managers considered those who focused on task completion as neglecting good care. This deputy manager described how some staff became further task oriented as a result of being told off for finding the balance difficult:

“*Yes, because then it becomes oh, my God, what should I do? I’m not going to spend time with this person because I need to do that, otherwise I’m going to be told off. It’s always this, kind of, I’m-going-to-be-told-off kind of attitude, which obviously damages the way you work a lot. Because then it becomes, like, obviously task based, you know […] you need to make sure that this person is being checked regularly… write it down on the paper because otherwise they’re going to tell you off.” (Deputy manager 1)*

There was acknowledgment from management of these organisational constraints, but the response was simply feeling that little could be done about it:

“*[…] sometimes it can be challenging because if the budget doesn’t meet, or if you go a bit over, then the staff needs to be reduced, and there is always the stage where the needs of the residents take second place because you need to reduce the staff and that becomes a problem.” (Deputy manager 1)*

Those staff who did not seem to be able to be person-centred as well as manage tasks were seen to be at fault. Both managers and care staff frequently mentioned that some staff simply did not have the right personalities, qualities or motivations for doing the job:

“*I said, if you don’t feel that you are in the right place just change job, you know what I mean? This is not the job for you. Nobody’s going to blame you for that, you know. But there are plenty of jobs there, where you can easily manage things in a different way. You can also slam the door behind you. Nobody’s going to care.” (Care assistant 2)*

At worst, the difficulties carers experienced were seen as individual failures and incompetence by managers:

“*We had one gentleman who used to shout. He never raised a finger, but he shouts, and he made a night carer lock herself in the office. And the question was, well, you’ve got 19 other residents on this unit. It’s only you on the unit at the moment, and you’ve just locked yourself in the office. Where’s the emergency buzzer? Where is…? You now, if you’re that scared, what are you doing?” (Deputy manager 2)*

When staff came up with innovative ideas that worked their creativity was only occasionally recognised. This manager, when asked if staff feel valued during the job, spoke of the lack of appreciation for those who successfully managed difficult situations:

“*No, not often, unless you’re successful, unless you have that lucky moment where you actually intervene and for whatever reason you actually manage to distract a person. And then everybody… oh, excellent, you’ve done it. Otherwise no; it tends always to be, well, you should have done this, you should have thought that, why you didn’t think about it, why you didn’t think about this, and most of the time, even if nobody tells you anything, you just had a horrible afternoon and nobody’s even recognising that, so no, I would say.” (Deputy manager 1)*

### Subtheme: Views of nurses

Of the two nurses interviewed across different homes, one nurse spoke of a more supportive and valuing work environment:

*“We support them and we support each other in many ways… if you’re in the office you leave the office and go and lend your hand*.” *(Nurse 1)*

However, we found that nurses expressed similar concerns over the workload and pressures of routinized care, and the effects on their ability to perform good care:

“*Up to resources really, whether we have enough human or otherwise… If, for instance, the suggestion or recommendation that you spend about 20 or 30 minutes doing one-to-ones, in practice it’s not… it becomes very difficult. And that’s beyond our control.” (Nurse 1)*

“*You’re stressed and whatever, you know, and you may say something that you know you shouldn’t say, or you may raise your voice at a resident, which you know you shouldn’t do, but at that moment, you’re thinking, oh, no, again.” (Nurse unit manager 1)*

We found that whilst the nurses did not share as strong views on not being treated in a dignified manner, they did struggle with a sense of value. This nurse agreed with how difficulties managing agitated behaviours could make impact negatively on their performance and carer identity to managers or relatives:

“*It does affect us if we are not able to manage certain behaviours it would appear that maybe we are not doing our job well.” (Nurse 1)*

However, this small sample makes it difficult to attribute these views to their professional role.

#### Views between roles

Some views about the perceived differences between nurses and care staff gave insight into how their role might impact on their views. For some care staff, they were in it together.

“*Say if I had a grievance with a nurse on the suite I will actually go up to them and say something to them now because I think to myself we’re all doing the same job*, *I’m just not giving medication*.*” (Care Assistant 2)*

However, some carers and management expressed a concern that nursing staff could be too medical in their approach. This manager spoke about a difficulty in hiring ex-nurses as carers for those reasons:

“*I think we suffer a little bit, maybe a lot, from that sort of nursing background in care…you know these are your jobs, these are your tasks allocations, get them done. And the relationship with the patient definitely came way down the list somewhere” (Home Manager 2)*

In contrast, one of the nurses expressed frustration over a perceived lack of standards or commitment from other non-professional carers:

“*I can’t understand sometimes why they’ve chosen to do the job because they don’t show any inclination towards caring or getting to know the person so that you can then treat them in the best way” (Nurse Unit Manager 1)*

… and they also mentioned the difficulties of working with 'disillusioned' staff:

“*Normally I would … try to boost them and things like that, but it’s becoming more difficult because… they’re not working to the standards that would like, so, I find that quite negative in a way because then you feel, you know, you’re not doing it, I’d like you to give more, but they are disillusioned as well (Nurse unit manager 1)*

There was a sense that these role conflicts could impact negatively on teamwork. However, there was also a general valuing of each other’s contribution to the team, and it seemed that their experiences were more closely aligned together than with management. Many of the issues affected staff of all levels, regardless of role.

## Discussion

The personhood of paid carers working with people living with dementia is an important and often neglected aspect of person-centred care. The views and experiences recounted in these interviews draw out a number of significant themes that may assist practitioners considering how to enhance the wellbeing of paid carers.

### Care staff not accepted as dignified persons in their own right

Whilst care staff are asked to implement person-centred care strategies and policies for care home residents, this approach is not necessarily extended to them. Care staff and management comments often indicated that staff were not accepted as dignified persons in their own right. Sometimes staff accepted this and at other times felt it contradicted their rights. However, staff consistently felt they had little control or influence over these working conditions. These findings suggest a distinct lack of support and respect for staff needs, in spite of the physical and emotional burdens of caring. This leaves them in danger of being viewed as depersonalised instruments of care labour, the objects of a management task force.

Whilst carers might feel physically abused by agitated residents, management denial of this and the subsequent requirement to accept it as part of the job without adequate support is depersonalising. It contradicts person-centred care principles, where care staff are required to attribute personhood to residents in order to care for them effectively. Care staff are expected and required to accept the behaviour of residents even if it is potentially detrimental to their wellbeing—any response would run the risk of blame or even potential disciplinary procedures. The contradiction between the implied personhood of residents and the denied personhood of care staff is stark. If such negotiations are difficult for better paid professional staff they are much more so for low paid 'unskilled' workers.

Although staff are asked to support the personal identities of residents, those carers whose caring values stem from their personal and cultural backgrounds can find themselves unable to incorporate them in their work, and are required to conform to a more operational form of care. At the same time care staff are required to engage, day-to-day, in intimate and familial relationships with residents, generally in the absence of family members. That they can have these relationships and experiences undermined and are themselves reduced to objects of care, or associated with negative media reports when real family visit, is depersonalising. Such conflicts are linked to staff burnout and job satisfaction [43], though the complexities of these interactions in regards to personhood warrants further investigation.

Despite widespread adoptions of person-centred cultures in dementia care, it seems that a genuine holistic culture change is still one level removed from the people who provide care. Findings suggest that staff are not being valued, and that this may be implicit within the core person-centred culture: their devaluing seen as part of the job and something to be put up with. Whilst staff may wholly carry out person-centred procedures, these do not acknowledge the inherent tensions and complexities of working in dementia care. Personhood when applied to staff does not appear to fit: it seems they are both ignored and have to put up with it.

### Moral work and labour–the identity of care staff

A developing theme from these interviews concerns a general lack of acknowledgment of the moral work of caring that occurs within formal care work; an oversight that can reduce the complex relationships of care work to a routinised process of work and which in turn challenges the self-worth of care workers.

Those working in formal care are assumed to be driven by a moral imperative to care [32]. Such moral work is important in helping to reduce the sense of abjection, vulnerability and helplessness that has been described in care homes, as well as maintain a person with dementia’s personhood as their infirmity increases: through social interaction, co-constructing care, and as professional care takes the place of traditional family support [44–46]. This moral work is also important in determining the self-value of carers; enabling them to frame their work as virtuous, despite poor conditions, the abject nature of the personal care performed, and care work’s historic associations with low status, unskilled work, once performed by pauper nurses in the workhouse infirmaries [32]. However, current care work is often procedural and task oriented, delivered by care companies which often prioritise profit. This conflict between the moral imperatives of care and the professional frameworks of labour results in a fundamental split identity for care workers [47].

Whilst those interviewed often displayed a genuine vocation for caring, they struggled to maintain a balance between the procedures of care work and organisational constraints and pressures, and their wish to genuinely care. Consequently, care staff felt out of control and pushed into a more instrumental form of care. That these conditions could ultimately threaten the quality of care and personhood of residents, diminishes staff ability to frame their work as dignified and virtuous. Concurrent findings have associated neglectful care behaviours with staff burnout [48]. Management responses typically contained a lack of understanding, blame and even doubts over carers’ abilities and motivations for the work. Reluctance by management to acknowledge problems that arise in caring might stem from worries that there are no solutions, or no good solutions. If care workers’ labour is simply measured in terms of the execution and manner of the tasks performed and they are not supported, opportunities for learning as well as dignity are denied. Attitudes among managers that good carers just get on with it, or have an innate awareness of how to care, both ultimately deny carers the support that comes from the acknowledgment that they are doing challenging, moral work and they have a right to support and training. Such issues seem inherently symptomatic of the current organisation, politics and delivery of formal care.

Person-centred attitudes and perceived competence in providing dementia care are consistently associated with care staff job satisfaction [49]. However, carers can be at odds with a procedural and accountable form of person-centred care, where oppressive institutional structures threaten their satisfaction and self-worth by limiting their ability to exercise rational choices and perform moral duties. Such findings may also contribute to understanding why randomised trials might fail to show effects for personally tailored activities of care [50]. Disparate interpretations of person-centred care have made the true meaning of the term to evaluate [18] and subsequently care staff can see person-centred care as an ideal, elusive, or feel sceptical about such theory in practice [51]. A need to oversee and make care accountable may become an end in itself: where the meeting of abstract standards supplants that of meeting people’s needs [52]. It may also neglect the reasons many carers do their job, caring because they choose to rather than being told to.

Whilst the majority of care staff views are from those who do not hold professional qualifications, it seemed that nurses too experienced similar conflicts over their desire and ability to care effectively in the care environment, suggesting the issue may go beyond simply having professional training—although their roles might distance them from some of the more depersonalising experiences. More research would be needed to investigate this further.

Frustrations regarding the different standards and approaches of ‘professionals’ and ‘non-professionals’ may be linked to dynamics within the UK care force. For example, those who may have worked as nurses in other places and contexts and who are now unable to may be experiencing a frustrating loss of professional status (such as that accorded to nurses) and identity, which is likely to add to their feelings of devalue. The lack of recognition of the skills of foreign professionals in this manner, who are often supporting themselves through lower status work, may be linked to greater stress, and there are calls for more attention in understanding race dynamics and promoting the rights of the migrant workers playing an important role in the UK care sector [53]. It may also be that those working as nurses (or indeed managers) hold onto their status as ‘professionals’, but that this leaves them feeling isolated from other care staff.

## Conclusions

Whilst training and support for paid care staff has become of increasing interest in dementia care, there has been a lack of acknowledgment of the personhood of paid care staff in dementia care. Caring is complex and challenging work, in which the skilled and moral work of carers is often unacknowledged and where carers can have their own needs and vulnerabilities subjugated to caring for those with dementia. It could be one of the contributing factors to the difficulty of establishing how person centred care can result in better outcomes. These interviews suggest that one factor might be that the contradictory way in which person-centred care is implemented in care homes [48] may relate to the unacknowledged personhood of staff, and consequent instrumentality, burnout and retention problems. There is a need to close the widening gap between an increasingly professionalised rhetoric and the everyday realities facing those who provide care [32]. We suggest that care staff are explicitly considered as part of quality standards and better supported by employer’s policy and practice. In this way, person-centred care can strive to be a philosophy that includes all those who work in dementia care.

### Limitations and suggestions for future research

Whilst we have set out the arguments for the value and acceptability of performing a secondary qualitative analysis in this area, there are also clear limitations of using this approach. The interviews did not focus explicitly on themes of staff personhood, and it is likely that some staff would be less spontaneous in offering their views on such issues. As not all of the data is relevant, this may limit the number of viewpoints represented, such that a greater sample size would may have been more preferable and therefore more illuminating. A greater sample size may also have helped to disentangle some of the issues surrounding differences in professional and non-professional roles and individual home cultures, in what is clearly a more complex picture than this data can reveal.

We argue that whilst these inherent limitations are present, this study raises an important subject that might not otherwise had been investigated. There is evidently considerable complexity to the issues raised and thus a corresponding need for a more direct research, both within this group and within wider settings in order to better understand the personhood of care staff. This may provide new considerations of care, regulation of conditions, and understandings on the nature of difficult care and abusive behaviours.
